# Chemical Atherogenesis: Role of Endogenous and Exogenous Poisons in Disease Development

**DOI:** 10.3390/toxics2010017

**Published:** 2014-01-20

**Authors:** Matthew K. Ross, Anberitha T. Matthews, Lee C. Mangum

**Affiliations:** Center for Environmental Health Sciences, Department of Basic Sciences, College of Veterinary Medicine, Mississippi State University, Mississippi State, MS 39762, USA; amatthews@cvm.msstate.edu (A.T.M.); lcm78@msstate.edu (L.C.M.)

**Keywords:** chemical atherogenesis, endogenous toxins, environmental pollutants, atherosclerosis, carboxylesterases, inflammation, oxidative stress

## Abstract

Chemical atherogenesis is an emerging field that describes how environmental pollutants and endogenous toxins perturb critical pathways that regulate lipid metabolism and inflammation, thus injuring cells found within the vessel wall. Despite growing awareness of the role of environmental pollutants in the development of cardiovascular disease, the field of chemical atherogenesis can broadly include both exogenous and endogenous poisons and the study of molecular, biochemical, and cellular pathways that become dysregulated during atherosclerosis. This integrated approach is logical because exogenous and endogenous toxins often share the same mechanism of toxicity. Chemical atherogenesis is a truly integrative discipline because it incorporates concepts from several different fields, including biochemistry, chemical biology, pharmacology, and toxicology. This review will provide an overview of this emerging research area, focusing on cellular and animal models of disease.

## 1. Introduction

Cardiovascular disease (CVD), hypertension, and obesity are overwhelmingly prevalent in western populations. Chemical atherogenesis, which initiates development of CVD, is an emerging field that describes how environmental pollutants and endogenous toxins collectively perturb critical molecular and cellular pathways that regulate lipid metabolism and inflammation, thus injuring cells found within the vessel wall. The term was originally coined by Ramos [1] and conceptualized the role of xenobiotics in the development of atherosclerotic plaques and progression of cardiovascular disease. A list of exogenous chemicals/environmental pollutants that are thought to contribute to atherosclerosis development is given in Table 1. Most of these compounds were shown to cause disease in animal models of atherosclerosis. Air pollution has gained significant interest as an etiological factor; however, due to limitations in the scope of the present review the reader is directed to a recent excellent review on this topic for more in depth information [2].

Chemical atherogenesis is analogous to the concept of chemical carcinogenesis in cancer research. However, despite growing awareness of the importance of environmental pollutants in the development of cardiovascular disease, the field of chemical atherogenesis can broadly include both exogenous and endogenous toxic compounds and the molecular, biochemical, and cellular pathways that become dysregulated by these poisons. This integrated approach is logical because exogenous and endogenous toxins often share the same mechanisms of toxicity [13]. Furthermore, because studies of human disease caused by chemicals in the environment are plagued by uncertainties regarding exposure, focusing on mechanisms by which endogenous chemicals contribute to the pathogenesis of atherosclerosis has a certain appeal because the issue of exposure to these chemicals and internal dose are unequivocal. Indeed, all mammalian cells are exposed to a barrage of endogenous toxins and many are considered to be atherogenic (see Table 2 for examples).

Atherosclerosis is a disease distinguished by two pathological characteristics: (i) deregulated lipid metabolism and (ii) uncontrolled inflammation [24]. Genetic and lifestyle factors are clearly important contributors to the pathological processes that underlie atherosclerosis development, and these issues have been widely reviewed [25]. In addition, environmental chemical pollutants have an important role in the initiation and progression of atherosclerosis [1]. As already stated, the important role of endogenous toxins in the development of atherosclerosis should also be considered in this context [13]. Because of the broad scope of the atherosclerosis field and wide swath of etiological factors that have been characterized, it is impossible to give an exhaustive review of this area and to characterize all environmental pollutants associated with this disease and to cite all relevant literature. Chemical atherogenesis is a truly integrative discipline because it incorporates concepts from several different fields, including biochemistry, chemical biology, cell biology, pharmacology, and toxicology. Therefore, this review will provide a brief overview of how endogenous and exogenous poisons can mechanistically contribute to the initiation and progression of atherosclerosis in animal and cellular model systems. Mechanisms by which chemicals injure cells that comprise the vessel wall will be integrated into the following sections below, which are devoted to atherogenesis, oxidative stress and NADPH oxidase, macrophage reverse cholesterol transport, the emerging role of the endocannabinoid system, and a final section devoted to cigarette smoke pollutants.

## 2. Atherogenesis

One of the key pathological features of atherogenesis is endothelial cell dysfunction [26]. Indeed, it is thought to be a prerequisite for atherosclerosis. When the endothelial cell layer becomes damaged circulating monocytes in the blood are recruited to the site of injury by chemokines and cytokines released by the injured endothelial cells, such as monocyte chemoattractant protein-1 (MCP-1). The rolling, tethering and diapedesis steps undertaken by activated monocytes along the endothelium have been well-described in several excellent reviews [24,26]. Following migration through the damaged endothelial cell layer and eventual recruitment into the vessel wall intimal space, the monocytes are exposed to an array of inflammatory and toxic molecules that induce differentiation. CD36 and SR-A are scavenger receptors upregulated during monocyte differentiation into macrophages. This is a critical phenotypic change because intimal macrophages are exposed to low-density lipoprotein (LDL) particles that have been chemically modified by oxidants and reactive lipid peroxidation products that are generated in the vessel wall. LDL particles play vital roles in bidirectional transport of lipids between the liver and peripheral tissues. They circulate in the blood but can migrate out of the circulation into the intimal space of vessel walls where they become entrapped by extracellular peptidoglycans. The entrapped LDL, which contains an internal cargo rich in cholesteryl esters and triacylglycerols surrounded by a phospholipid and apolipoprotein B (apoB) shell, is subsequently exposed to a host of oxidants and electrophiles that are liberated by endothelial cells, macrophages, and smooth muscle cells. As a result, the phospholipid and apoB shell is oxidatively modified yielding oxidized (ox)LDLs. One of the cardinal features of oxLDL is the presence of oxidized phosphatidylcholine (oxPC_CD36_) on its surface [16]. oxPC_CD36_ are detected in atherosclerotic plaques and in the circulation of hyperlipidemic subjects [27] and contain truncated sn-2 oxidized lipids that protrude like whiskers from the surface of oxLDL particles. oxPC_CD36_ is denoted with the subscript CD36 because it binds to the scavenger receptor CD36 with high affinity, thus enabling avid interactions between macrophage and oxLDL particles. Following the engagement of oxidized phospholipids with the CD36 receptors, the oxLDL particle is subsequently engulfed/phagocytosed by the macrophages. Upon internalization, the lipid-rich cargo of oxLDL is metabolized by a complex set of biochemical pathways resulting in accumulation of a large number of neutral lipid droplets within the cytoplasm, which accounts for the characteristic foamy appearance of lipid-engorged macrophages. These pathogenic cells are responsible for the initial fatty streaks that herald atherosclerosis [24].

**Figure 1:**
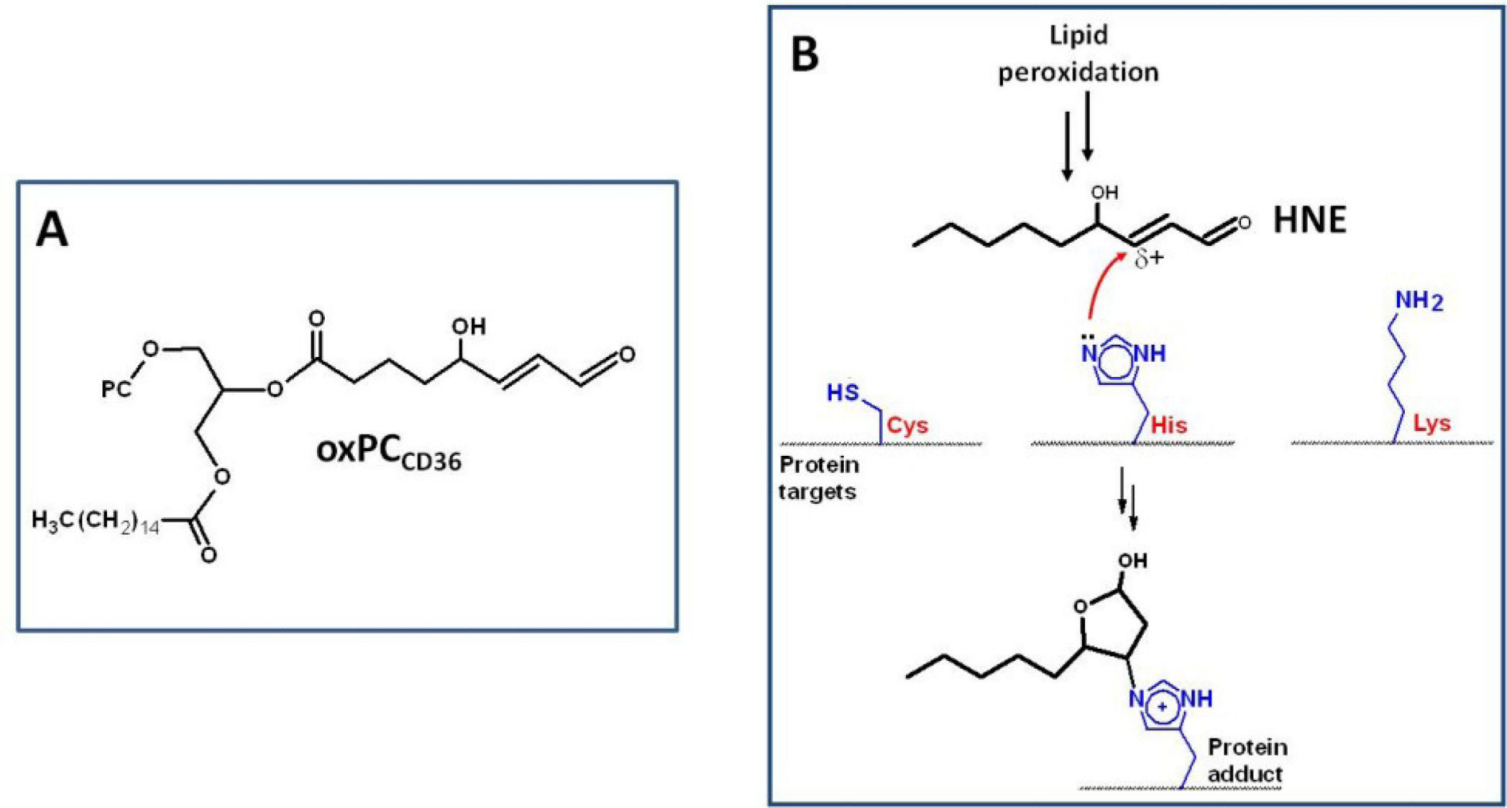
Structures of α,β-unsaturated electrophiles that can undergo Michael addition reactions with cellular nucleophiles. (**A**) Structure of oxPC_CD36_, a γ-hydroxyalkenal phospholipid analogue of 4-hydroxynonenal (HNE); (**B**) Lipid peroxidation generates the diffusible aldehyde HNE, which can modify amino-acid side chains in proteins. Shown is the reaction between histidine and HNE, although HNE can also react with cysteine and lysine residues.

oxPC_CD36_ is an electrophilic molecule that can covalently modify proteins (Figure 1). Incubation of a synthetic biotin-tagged oxyphospholipid probe with human plasma enabled the plasma proteins that chemically reacted with the electrophilic probe to be identified [28]. It was shown that apolipoprotein A1 (ApoA1), which is the core protein of high-density lipoproteins (HDL), was the most highly modified plasma protein. The biotin tag allowed the electrophile-modified proteins in plasma to be affinity purified, trypsinized, and the tryptic peptides analyzed by mass spectrometry. ApoA1-lipid electrophile adducts were mapped to His162. Adduction of ApoA1 by oxidized phospholipid can alter the beneficial functions of HDL, in particular its ability to ferry cholesterol from peripheral tissues to the liver [29]. In addition, oxidized phospholipids have been shown to be directly toxic to macrophages by inducing apoptosis [30].

**Figure 2:**
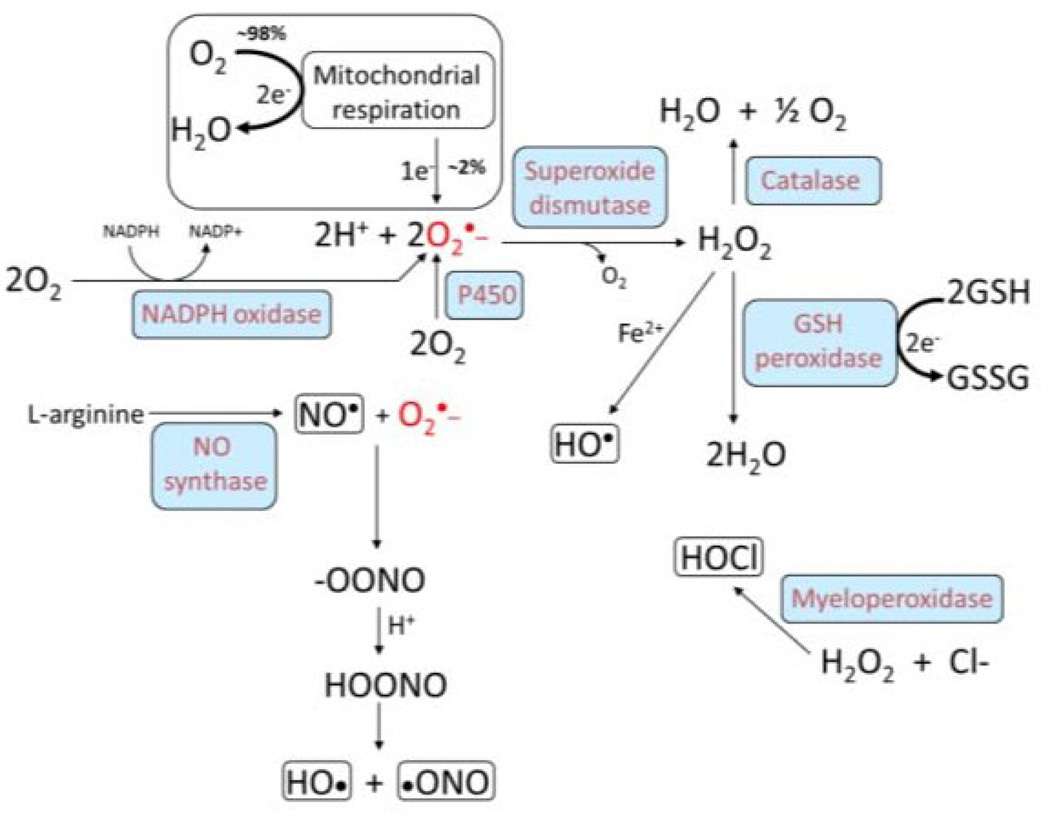
Redox chemistry important for the generation of reactive oxygen species during atherogenesis. Approximately 98% of the oxygen consumed in mitochondria is converted to water while 2% undergoes one electron reduction to superoxide. Redox enzymes are denoted in blue.

The source of the oxidants in the vessel wall that damage LDL particles is still a matter of debate, but NADPH oxidases, myeloperoxidases, lipoxygenases, and uncoupled nitric oxide synthases are strongly expressed in intimal macrophages and endothelial cells and are likely candidates (Figure 2). Lipid peroxidation of LDL by unpaired oxygen radicals causes a chain reaction of events that propagates the generation of oxyradicals and lipid peroxides, which leads to formation of electrophilic α,β-unsaturated aldehydes, such as 4-hydroxynonenal (HNE) and 4-oxononenal (ONE). Although HNE is a chemically reactive molecule it can diffuse through lipid membranes [31], and α,β-unsaturated aldehydes are toxic to endothelial cells [16]. HNE can modulate the expression of adhesion molecules such as intercellular adhesion molecule-1 (ICAM-1) [32], which tether circulating monocytes to the endothelium enabling inflamed monocytes to migrate into the vessel wall. HNE also covalently modifies amino-acid side chains in apolipoprotein B in LDL, further increasing the uptake of oxidized and chemically-modified LDL by both macrophages and smooth muscle cells and enhancing foam cell formation.

A critical feature of oxLDL uptake by macrophages via scavenger receptors is that it is an unregulated process. Because of the lack of negative feed-back mechanisms that shutdown oxLDL uptake, the cells become engorged with excess cholesterol. To counteract the increasing amounts of endoplasmic reticulum membrane-associated cholesterol in the cell and the resulting endoplasmic reticulum stress that activates apoptotic pathways [33], free cholesterol is esterified to fatty acyl groups by the enzyme acyl CoA:acyltransferase-1 (ACAT-1) and stored within biologically inert neutral lipid droplets [34]. However, this process can become overwhelmed and once a threshold level of unesterified cholesterol is reached the foam cells undergo apoptosis with subsequent macrophage efferocytosis (*i.e.*, the phagocytic clearance of apoptotic cells by neighboring macrophages). In early stages of atherosclerosis, the process of efferocytosis is relatively efficient; however, it can become defective at later stages. This causes the phagocytic macrophages to undergo secondary necrosis, which only further exacerbates the inflammatory and oxidative environment of the vessel wall.

## 3. Oxidative Stress, NADPH Oxidase, and Atherosclerosis

The mechanistic relationship between xenobiotic exposures and increased atherosclerotic risk is complex. However, a central mechanism is thought to be an elevation in reactive oxygen species and resulting oxidative stress (Figure 2), which is often due to inappropriate activation of key enzymes.

Increased levels of oxyradicals in cells and tissues often hinge on inappropriate activation of NADPH oxidase, a holoenzyme responsible for catalyzing the synthesis of superoxide in multiple cell types including endothelial cells and macrophages. NADPH oxidase could be activated in cells upon exposure to xenobiotics including dieldrin and lindane, which are chlorinated cyclodiene insecticides widely used in the 1950s and 1960s [35,36]. The NADPH oxidase holoenzyme in macrophages is composed of two membrane-bound subunits, Nox2 (Nox4 in endothelial cells, Nox1 in smooth muscle) and p22phox, as well as several cytosolic regulatory subunits, including p40phox, p47phox, p67phox, Rac1, and Rac2. During enzyme activation cytosolic subunits are stimulated to translocate to the cell membrane where they assemble with the Nox2/p22phox heterodimer. Superoxide anion is produced when the multi-subunit complex transfers electrons from NADPH to molecular oxygen. Activation of NADPH oxidase can lead to a rapid accumulation of superoxide-derived reactive oxygen species that are capable of crossing lipid membranes via anion channels (O_2_·^−^) and passive diffusion (H_2_O_2_) thereby reacting with a wide range of cellular constituents. Oxyradical flux may be directly linked to the progression of atherosclerotic disease via peroxidation of low-density lipoprotein (LDL) [16,36]. There is also evidence indicating that arachidonic acid, which is liberated by phospholipase A_2_, is the primary trigger molecule responsible for induction of NADPH oxidase activity subsequent to organochlorine insecticide exposures [37]. Arachidonic acid has been demonstrated to modulate NADPH oxidase subunit assembly, and therefore activity, by exposing Src homology 3 domains on the p47phox subunit, which is necessary for efficient interaction with p22phox. The association of regulatory subunits and the concurrent phosphorylation of p47phox and/or p67phox by protein kinases, including p38 MAPK and PKC-δ (which may both be activated by arachidonic acid), act in concert to stimulate NADPH oxidase activity [38].

**Figure 3:**
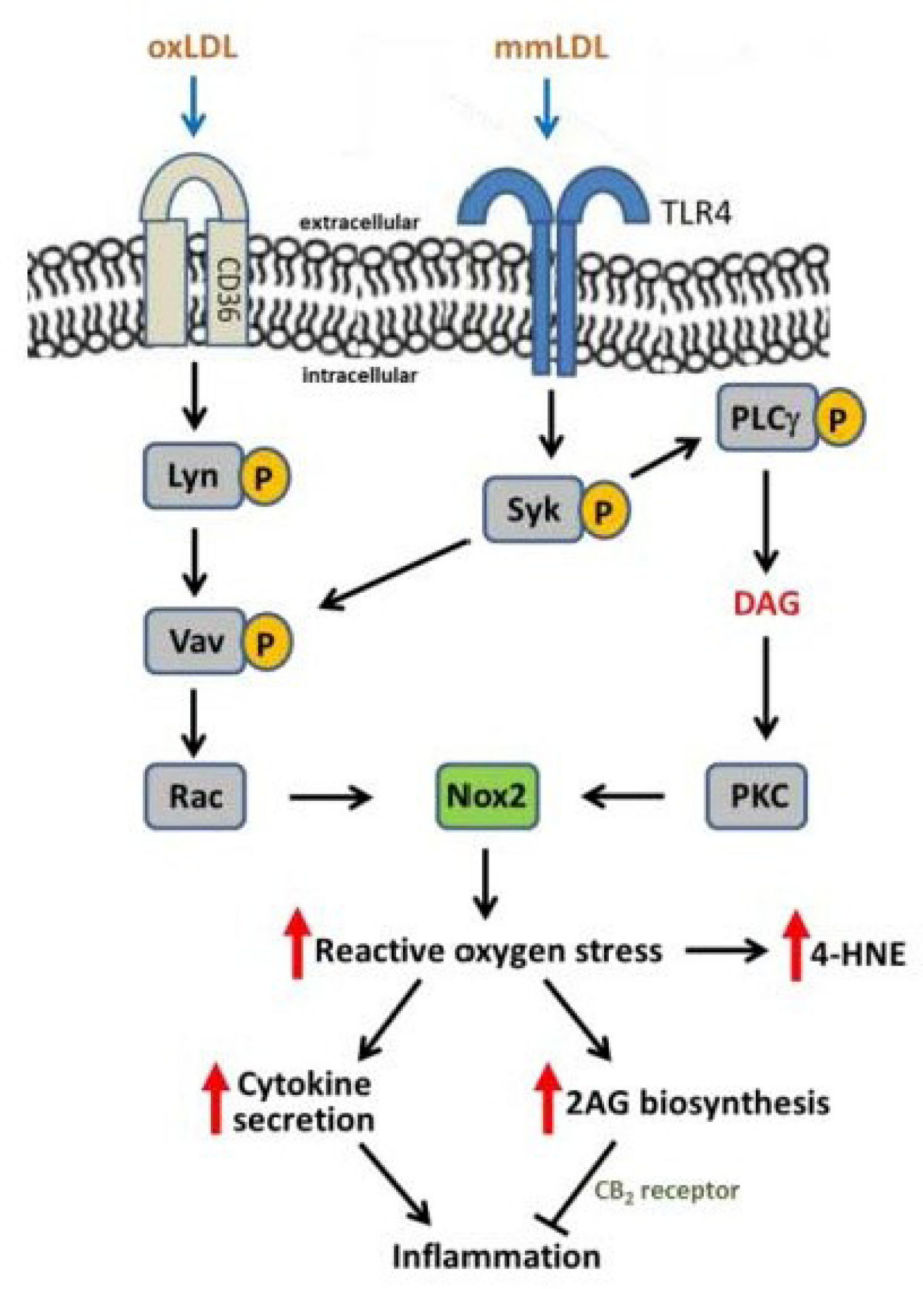
Signaling pathways in macrophages responsible for activation of NADPH oxidase caused by exposure to oxidized (ox)LDL and minimally modified (mm)LDL. Abbreviations: *CD36*, scavenger receptor; *TLR4*, toll-like receptor 4; *Lyn* and *Syk*, Src family tyrosine kinases; *Vav*, guanine nucleotide exchange factor; *Rac*, GTP-binding protein; PLCγ, phospholipase Cγ; DAG, diacylglycerol; PKC, protein kinase C; Nox2, NADPH oxidase catalytic subunit; 4-HNE, 4-hydroxynonenal; 2AG, 2-arachidonoylglycerol; CB2, cannabinoid receptor 2.

OxLDL is another type of endogenous activator of NADPH oxidase and the catalytic subunit Nox2 is a central node in the biochemical pathways activated by oxLDL (Figure 3). OxLDL can activate CD36 scavenger receptor-evoked signal transduction pathways in macrophages, leading to the stimulation of NADPH oxidase [39]. The subsequent production of superoxide can potentiate the buildup of oxLDL in a positive feedback mechanism, eliciting further increases in superoxide production, LDL oxidation, and atherosclerosis progression [35]. Macrophages express Nox2 abundantly, which accounts for why these cells generate large amounts of ROS [40]. It has been shown that human macrophage-like U937 cells are more responsive than human THP-1 cells to oxLDL with respect to reactive oxygen species generation, which is attributed to a greater number of CD36 receptors present in U937 cells compared to THP-1 cells [41]. Deletion of Nox2 in mice decreased the extent of atherosclerotic lesions in ApoE^−/−^ mice administered a high fat diet [42]. From a clinical point of view, Drummond *et al*. [43] indicated that premenopausal females have a lower propensity to develop CVD than males, postmenopausal women, and ovariectomized females because elevated levels of estrogens actually help to dampen NADPH oxidase activity. Therefore, development of Nox2 inhibitors is emerging as an attractive strategy to block atherogenesis and atherosclerotic progression [43].

NADPH oxidase-derived superoxide has also been shown to modulate the vascular endothelial growth factor A (VEGF-A) signaling pathway, suggesting a link between increased NADPH oxidase activation and increased endothelial migration and proliferation that occurs during atherosclerosis [35,44]. In addition, high levels of 8-*iso*-PGF_2α_, a stable prostaglandin-like molecule called isoprostane that is formed during free-radical peroxidation reactions involving arachidonic acid found in lipid bilayers, may also contribute to atherosclerosis development [45,46]. 8-*iso*-PGF_2α_ can enhance the adhesion of monocytes to the vascular endothelium following its ligation of endothelial-cell surface thromboxane receptors [47], thus resulting in the upregulation of endothelial adhesion molecules and accelerating monocyte/macrophage infiltration of the arterial intima.

With regard to NADPH oxidase function, an important homeostatic effect of the vasoprotective molecule prostacyclin (PGI_2_), which is produced by endothelial cells, is its ability to tonically suppress NADPH oxidase activity in endothelial cells via autocrine/paracrine signalling, thereby protecting against endothelial cell dysfunction caused by increased oxidative stress and inflammation [48]. This is due to the ability of PGI_2_ to inactivate protein kinase-δ, which is responsible for phosphorylation of the cytoplasmic subunit p47phox. Phosphorylated p47phox rapidly translocates to the Nox2-p22phox catalytic subunits in plasma membranes, thus forming the active NADPH oxidase holoenzyme complex. Interestingly, it was shown that exposure to tobacco smoke within the context of inflammation, where IL-1β levels were elevated, caused a reduction in PGI_2_ levels and a subsequent derepression of NADPH oxidase activity [48]. The increased flux of superoxide caused microsomal prostaglandin E_2_ synthase (mPGES) to be upregulated, which raised pro-inflammatory PGE_2_ levels, thereby establishing a feed-forward mechanism that enhanced inflammation and endothelial dysfunction [48].

## 4. Macrophage Reverse Cholesterol Transport

Cellular mechanisms to reduce the burden of cholesterol in vessel wall macrophages and other peripheral cells are limited. Although not a very efficient reaction pathway, cholesterol can be oxidized by cytochrome P450s to yield oxysterols for two purposes. First, it makes cholesterol more water soluble and excretable; second, it produces bioactive oxysterols that bind to nuclear receptors, such as liver × receptors (LXR-α and -β), which are ligand-activated transcription factors that regulate the expression of genes that encode cholesterol transporters and other genes that regulate cholesterol homeostasis [49]. For example, CYP27A1 oxidizes free cholesterol yielding 27-hydroxycholesterol [50], an oxysterol with high affinity for LXR. LXR controls the expression of *Abca1* and *Abcg1* genes, which encode ATP-binding cassette transporters (ABCA1 and ABCG1, respectively) that efflux non-esterified cholesterol from the macrophage plasma membrane onto extracellular ApoA1 and mature high-density lipoproteins (HDLs) [49]. This process is termed macrophage reverse cholesterol transport (RCT) and it is the mechanism by which cholesterol is moved from peripheral tissues, including vessel wall macrophages, into the circulation for eventual disposal in the liver [19]. A key step in macrophage RCT is the mobilization of cholesterol stored as cholesteryl esters in lipid droplets found in macrophages. This process is catalyzed by a neutral cholesteryl ester hydrolase; however, the identity of the hydrolase responsible for this reaction is highly controversial. One candidate is a xenobiotic hydrolase termed human carboxylesterase 1 (CES1) [51,52]. This enzyme is strongly expressed in both primary human macrophages and macrophage cell lines, including THP1 monocyte/macrophage cells. The overexpression of CES1 in THP-1 macrophages was shown to cause a marked increase in the rate of cholesterol efflux *in vitro* [53]. Moreover, macrophage-specific expression of human CES1 in mice enhanced the rate of RCT in the atherosclerotic-prone highfat diet-fed *Ldlr*^−/−^ mouse model and decreased the extent of atherosclerotic plaques [54]. Consistent with these findings, inhibition of CES1 activity in THP-1 foam cells caused by exposure to chemical inhibitors, such as organophosphorus insecticides, led to a significant increase in intracellular cholesteryl ester levels [55]. The active-site serine in CES1 reacts extremely rapidly with organophosphorus insecticides, exhibiting second-order inactivation rate constants (*k*_inact_/*K*_i_) ~10^6^–10^7^ M^−1^s^−1^ [56]. Thus, CES1 is a very sensitive target for environmental toxicants, such as OP pesticides, and its inhibition may be detrimental to health.

Interestingly, macrophage-specific CES1 transgenic *Ldlr*^−/−^ mice also exhibited improved glucose tolerance and insulin sensitivity, which was accompanied by reduced inflammatory mediator profiles when compared to non-transgenic controls [57]. These findings were apparently due to repression of NFκB and AP-1 transcription factor activities in adipose tissue macrophages of CES1 transgenic mice, which was associated with reduced macrophage cholesterol levels compared to non-transgenic controls. This finding was also consistent with results indicating that macrophages derived from *Abca1*^−/−^ mice produced higher levels of pro-inflammatory mediators compared to wildtype macrophages following stimulation [58]. *Abca1*^−/−^ macrophages had higher concentrations of free cholesterol than the wildtype cells. Thus, *in vitro* and *in vivo* studies point to an important role for CES1 in cholesteryl ester hydrolysis in macrophages, which could have important implications for the development of metabolic syndrome and atherosclerosis.

Further development of atherosclerotic disease is associated with a thickening of the intimal region due to the accumulation of macrophage foam cells and smooth muscle cell migration and proliferation within the intima [26]. Activation of matrix metalloproteinases (MMPs) subsequently causes degradation of collagen matrix leading to the thinning of the fibrous cap that covers the atheroma, thus setting the stage for the development of a “vulnerable plaque” that can rupture causing an acute myocardial infarction event.

## 5. Endocannabinoid System and Atherosclerosis

Accumulating evidence supports the idea that atherosclerosis is associated with endothelial dysfunction, hypertension, hyperlipidemia, and elevated concentrations of reactive oxygen species (ROS). In addition to these factors, an overactive endocannabinoid (EC) system may also contribute to atherogenesis. The EC system is comprised of several components, including G-protein-coupled cannabinoid receptors (CB_1_ and CB_2_), which modulate adenylate cyclase activity; arachidonoyl-containing ligands (2-arachidonoylglycerol, 2-AG; anandamide, AEA); biosynthetic enzymes (e.g., DAGLβ for 2-AG synthesis), and enzymes that degrade endocannabinoids (e.g., monoacylglycerol lipase, fatty acid amide hydrolase, and cyclooxygenase-2) [59]. The endocannabinoids are endogenously produced bioactive lipids with potent biological activities that are elicited following ligation of cannabinoid receptors. CB_2_ was previously thought to be expressed only in immune and hematopoietic cells, whereas CB_1_ was considered to be limited to the central nervous system. However, CB_1_ was subsequently found in peripheral tissues, including human coronary artery endothelial and smooth muscle cells [60], whereas CB_2_ is also found in myocardium, human coronary endothelial cells, smooth muscle cells, brain, and liver [59]. These findings suggest that the EC system plays a vital role in the modulation of the immune system in the context of atherosclerosis. Studies indicate that the EC system has an important role in vascular homeostasis and perturbation of the EC system may contribute to disease. Recent findings in animal models indicated that CB_1_ receptor antagonism [61,62] or CB_2_ receptor activation using agonists [63,64] were both atheroprotective strategies and reduced atherosclerosis. For example, a study by Zhao *et al*. [65] using ApoE^−/−^ mice and WIN 55212-2, a non-specific cannabinoid receptor agonist, demonstrated that accumulation of macrophages in the vessel wall intima was reduced and expression of inflammatory genes in plaque lesions was decreased. Interestingly, the effects elicited by WIN 55212-2 were abrogated by the addition of AM630, a CB2 antagonist. Further, this study showed a decrease in cytokines TNF-α, IL-6 and MCP-1 in the plaque lesions. Another study suggested that JWH-133, a CB_2_ agonist, reduced superoxide generation and increased ERK 1/2 and STAT3 phosphorylation, inhibited chemotaxis initiated by TNF-α, and upregulated CD18/CD11b (CR3) on neutrophils [59]. This last finding suggested that apoptotic inflammatory cells within the vasculature could be cleared by neutrophils following upregulation of CR3, a surface receptor on neutrophils. Consequently, efficient phagocytic clearance of lipid-laden foam cells can promote reduced inflammation and plaque formation. Activation of CB_2_ receptors can attenuate TNF-α and other inflammatory responses, whereas CB_1_ receptors are instrumental in the production of pro-inflammatory mediators. Thus, increasing CB_2_ receptor expression may render a cardioprotective effect.

With regard to endocannabinoids, AEA was shown to trigger activation of several stress kinases, including p38, Jun amino-terminal kinase–mitogen activated protein kinase (JNK-MAPK), extracellular signal-regulated kinase 1 and 2 (ERK 1/2), which can significantly impact cellular phenotype. Rajesh *et al*. [60] demonstrated that NADPH oxidase activity in human coronary artery endothelial cells could be attenuated by a CB_2_ agonist. In a year-long study in mice using rimonabant (CB_1_ specific antagonist), Sugamura [62] provided evidence that atherosclerotic lesions decreased in ascending abdominal aorta following treatment with rimonabant when compared to vehicle controls. Acceleration of reverse cholesterol transport due to enhanced cholesterol efflux via ABCA1 and ABCG1 and subsequent reduction in atherosclerosis was also demonstrated following pharmacological blockade or genetic deletion of the CB_1_ receptor [62,66]. Treatment of primary murine macrophages and macrophage cell lines with oxLDL enhanced the biosynthesis of the endocannabinoid 2-AG in a concentration dependent manner [67]. 2-AG is biosynthesized from the lipid precursor diacylglycerol (DAG), which is produced by the action of phospholipase Cβ on phosphatidylinositol, and DAG is subsequently hydrolyzed by diacylglycerol lipase (DAGL) yielding 2-AG in response to extracellular stimuli, including angiotensin II, thromboxane A2, platelet activating factor (PAF), bradykinin, serotonin, glutamate, and acetylcholine [68]. Gao *et al*. [69] used DAGL^−/−^ knockout mice to show that 2-AG biosynthesis was markedly attenuated following stimulation. Thus, toxicants that stimulate or inhibit DAGL activity may significantly alter endocannabinoid tone, which could subsequently affect physiological mechanisms in a variety of contexts. Furthermore, we discovered that CES1 and PPT1 are the main enzymes responsible for the hydrolysis of 2-AG in intact human THP-1 macrophages [70,71], and that treatment of THP-1 foam cells with OP toxicants significantly enhanced the levels of 2-AG because of CES1 inhibition [70]. It is possible that inactivation of CES1 by environmental toxicants can hyperactivate the endocannabinoid system in macrophages due to elevated 2-AG levels, which may impact cellular phenotypes. We are currently pursuing this concept.

## 6. Cigarette Smoke: A Modifiable Risk Factor

Cigarette smoke contains thousands of toxic chemicals and epidemiological evidence unequivocally shows that cigarette smoking is a major modifiable risk factor for the development of heart disease [72], although definitive molecular mechanisms are only just emerging. For example, cigarette smoke extract (CSE) can acutely increase the risk of myocardial infarction by raising levels of the bioactive lipid PAF in the circulation, which can markedly increase thrombotic risk due to platelet activation in the vessel lumen [73]. Interestingly, in this study cigarette smoke components were shown to inactivate the PAF hydrolase found in endothelial cells, thereby mechanistically accounting for the increase in levels of its cognate substrate, PAF. ApoE^−/−^ mice that were exposed for 21 and 42 days to 30 mg/m^3^ second-hand tobacco smoke exhibited markedly increased plaque lesion areas within the aortic sinus as compared to filtered air-exposed ApoE^−/−^ mice [74]. Mechanisms to account for this observation included the possibility that cigarette smoke components inhibited the activity of superoxide dismutase 2 (SOD2), which is responsible for detoxifying superoxide anion [74]. This suggested that a build-up of superoxide enhanced oxidative stress within artery walls, thus contributing to atherogenesis. Another deleterious consequence of high concentrations of superoxide is its ability to react with the important vasodilatory molecule, NO, thereby depleting the levels of this important molecule and also yielding a highly reactive molecule called peroxynitrite (^−^OONO) that can damage cellular constituents. Peroxynitrite can covalently modify the tyrosine amino acids in proteins, as indicated by the increased levels of 3-nitrotyrosine within the vessel wall [74]. Further exacerbating this situation is the possibility that pollutants found in cigarette smoke can inhibit the activity of endothelial nitric acid synthase (eNOS), thereby further reducing levels of the vasoprotective NO molecule. Moreover, because NO *per se* is so vital to inhibition of platelet aggregation and inflammation, its depletion during conditions of oxidative stress and eNOS uncoupling (when O_2_·^−^ is generated instead of NO) is highly detrimental to vascular health [75].

Acrolein is a well-studied component of tobacco smoke and also an endogenous lipid peroxidation product. Exposure to acrolein has been associated with acute myocardial infarction [76]. It is a reactive aldehyde that causes the adduction (*i.e.*, chemical modification) of amino acids in proteins, such as histidine, cysteine and lysine. For example, acrolein can covalently react with lysine residues in apoB of LDL particles, which leads to their uptake by macrophages and enhanced foam cell formation [77,78]. Treatment of THP-1 macrophages with acrolein *in vitro* caused an increased expression and activity of MMP-9 [79], suggesting a mechanism by which acrolein contributes to development of vulnerable plaques. This effect was dependent on production of ROS because antioxidants and xanthine oxidase inhibitors could block acrolein-evoked MMP-9 expression. Increased expression of MMP-1 was also observed in endothelial cells following exposures to CSE or acrolein, which was attributed to inhibition of mammalian target of rapamycin (mTOR) by the CSE [80]. Furthermore, tissue inhibitor of metalloproteases-3 (TIMP-3), a major regulator of angiogenesis, was significantly downregulated by CSE and acrolein. Other studies have shown that stimulation of J774A.1 macrophages with either acrolein or 4-hydroxynonenal resulted in the increased biosynthesis of proinflammatory eicosanoid LTB_4_, which was associated with increased 5-lipoxyenase expression [81,82]. LTB_4_ is known to increase the expression of MMP-9 [83]. Similarly, treatment of human coronary SMCs with either oxLDL or HNE enhanced the rates of production of MMP-1 [84].

## 7. Conclusions

CVD in its various forms is the number one killer in the world and atherosclerosis accounts for a significant fraction of CVD cases. Cellular components of the vascular wall can be injured by endogenous and exogenous chemicals, leading to development of atherosclerosis in humans and experimental models. Because antioxidant interventions have yielded disappointing results with regard to CVD outcomes, elucidating the cellular and molecular mechanisms that account for how chemicals injure vascular cells will provide insights into new pharmacological intervention strategies to halt chemical atherogenesis. For example, NADPH oxidase appears to be an attractive enzyme to inhibit using small molecules because it is a primary source of reactive oxygen species in the vessel wall. In addition, compounds that reduce the concentration of electrophilic α,β-unsaturated aldehydes by either direct covalent reactions or the induction of detoxication enzymes is another approach that may prove fruitful. Fundamental understanding of the processes that contribute to chemical atherogenesis will have a significant impact on human health.

## Figures and Tables

**Table 1:** Exogenous chemicals/pollutants associated with atherosclerosis development.

Pollutant	References
Acrolein	[3]
Allylamine	[4]
Arsenic	[5]
Benzo(a)pyrene, other PAHs	[6]
Bisphenol A	[7]
PCBs	[8]
Cigarette smoke constituents	[9]
Vinyl chloride	[10]
Air pollutants (particulate matter, ozone, and NO_*x*_)	[11,12]

**Table 2:** Endogenous chemicals associated with atherogenesis.

Compound	References
oxPAPC_CD36_	[14]
4-Hydroxynonenal	[15]
4-Oxononenal	[16]
Reactive oxygen/nitrogen species (O2·^−^,·OH,·NO, H_2_O_2_)	[17]
Saturated fatty acids	[18]
Cholesterol	[19]
Oxysterols	[20]
Isoprostanes	[21]
Eicosanoids	[22]
Lipopolysaccharide (LPS)	[23]
